# *SIRT1* Polymorphisms Associate with Seasonal Weight Variation, Depressive Disorders, and Diastolic Blood Pressure in the General Population

**DOI:** 10.1371/journal.pone.0141001

**Published:** 2015-10-28

**Authors:** Leena Kovanen, Kati Donner, Timo Partonen

**Affiliations:** 1 Department of Health, Mental Health Unit, National Institute for Health and Welfare (THL), Helsinki, Finland; 2 Institute for Molecular Medicine Finland (FIMM), University of Helsinki, Helsinki, Finland; Tulane School of Public Health and Tropical Medicine, UNITED STATES

## Abstract

*SIRT1* polymorphisms have previously been associated with depressive and anxiety disorders. We aimed at confirming these earlier findings and extending the analyses to seasonal variations in mood and behavior. Three tag single-nucleotide polymorphisms (SNPs) were selected to capture the common variation in the *SIRT1* gene. 5910 individuals (with blood sample, diagnostic interview, self-report of on seasonal changes in mood and behavior) were selected from a representative Finnish nationwide population-based sample. Logistic and linear regression models were used to analyze the associations between the SNPs and depressive and anxiety disorders, metabolic syndrome (EGIR criteria) and its components, and health examination measurements, Homeostasis Model Assessments, and diagnoses of type 2 and type 1 diabetes. *SIRT1* rs2273773 showed evidence of association with seasonal variation in weight (*C*-allele, OR = 0.85, 95% CI = 0.76–0.95, p = 0.005). In addition, our study gave further support for the association of *SIRT1* gene with depressive disorders (rs3758391) and diastolic blood pressure (rs2273773).

## Introduction

Many recent studies have focused on sirtuin 1 (SIRT1) in relation to metabolism, insulin resistance, cancer, and longevity [1–3]. SIRT1, which is a histone deacetylase, participates through its deacetylase activity for tens of substrates in the coordination of a range of cellular functions, such as cell-division cycle, response to DNA damage, apoptosis, and autophagy. SIRT1 is also a sensor of the cytosolic housekeeping redox reaction of nicotinamide adenine dinucleotide that is measured with the ratio of the oxidized and the reduced forms, and that is changed by glucose deprivation and the metabolic changes under caloric restriction or fasting. There is an earlier report on *SIRT1* in metabolic syndrome, where there was no significant association [4].

So far, genetic variations in *SIRT1* have been associated with depressive [5] and anxiety [6] disorders. In an elegant study, both common and rare variations in *SIRT1* in humans were found to associate with the increased odds for anxiety disorders at large [6]. The study also demonstrated that in mice SIRT1 increases anxiety by deacetylating the brain-specific helix-loop-helix transcription factor, nescient helix loop helix 2 (NHLH2), which increases its activity on the monoamine oxidase A (*MAOA*) promoter. As the MAOA enzyme degrades serotonin and dopamine, the increased enzyme activity leads to reduced serotonin and dopamine levels in the brain, especially in those regions related to regulation of mood and emotions, and thereby to increased depression and anxiety [7–9].

Further, *SIRT1* variants have been associated with depressive disorder [5], but not with bipolar disorder [10]. However, during a depressive episode due to major depressive disorder or bipolar disorder, the mRNA levels of sirtuin isoforms in peripheral white blood cells, are lowered whereas the levels of those mRNAs in a remissive state are equal to those in healthy controls [11]. Here, it is of note that 10–20% of patients with recurrent major depressive disorder and 15–22% of those with bipolar disorder have the seasonal pattern for mood disorder, or seasonal affective disorder [12].

It appears that not only mood and behavior, but also the components, or risk factors, of the metabolic syndrome of the individual do fluctuate over the year. The increase in metabolic syndrome prevalence is mainly due to the increases in blood pressure and glucose during the winter, and the seasonal variation in metabolic syndrome prevalence associates with insulin resistance being increased from the extent of mild to moderate [13,14].

One aim of our current study was to confirm, as far as *SIRT1* is concerned, the earlier findings that have demonstrated associations of sirtuins with depressive and anxiety disorders. Another aim of our current study was to extend the exploration of associations of *SIRT1* to concern those with the seasonal variations in mood and behavior, metabolic disorder, and relevant health examination measurements. Here, we report associations to seasonal variation in weight, depressive disorders and diastolic blood pressure.

## Materials and Methods

### Subjects

The subjects were selected from the national Health 2000 survey [15] of Finnish population aged 30 years and older (n = 8028) living in mainland Finland that was approved by the ethics committees of the National Public Health Institute and the Helsinki and Uusimaa Hospital District. All participants provided a written informed consent. The selection (n = 5910) included individuals who had given blood samples, taken part to the Munich-Composite International Diagnostic Interview (M-CIDI) [15] and filled in the self-report on seasonal changes in mood and behavior adapted from the Seasonal Pattern Assessment Questionnaire (SPAQ) [16].

### Phenotypes

Depressive disorders (major depressive disorder, dysthymia) and anxiety disorders (panic disorder w/o agoraphobia, generalized anxiety disorder, social phobia, agoraphobia) without hierarchy criteria were assessed using M-CIDI, a valid and reliable instrument for the assessment of depressive, anxiety and alcohol use disorders yielding diagnoses according to Diagnostic and Statistical Manual of Mental Disorders, Fourth Edition (DSM-IV) [16]. The controls did not have any diagnosis of mental disorders nor met any sub-threshold criteria as assessed with the M-CIDI.

The participants filled in a questionnaire of lifetime seasonal variations in mood and behavior adapted from SPAQ [17]. The six items of sleep length, social activity, mood, weight, appetite, and energy level were scored from 0 to 3 (none, slight, moderate or marked change) rather than from 0 to 4 (none, slight, moderate, marked or extremely marked change), with the sum or global seasonality score (GSS) then ranging from 0 to 18. The psychometric properties of this modified questionnaire have been reported to be good [18]. In this study, dichotomous variables (no variation, variation) were computed for the six items.

Routine fasting laboratory tests included the concentrations of blood glucose, serum insulin, serum total cholesterol, triglycerides, low-density lipoprotein (LDL) cholesterol, and high-density lipoprotein (HDL) cholesterol. The Homeostasis Model Assessment (HOMA) insulin resistance and beta-cell function indexes were computed. Blood pressure, height, weight, and waist circumference were measured. Body-mass index (BMI) was calculated (as kg per m^2^). Diagnosis of type 2 diabetes and that of type 1 diabetes were assessed on the basis of all available data collected for the health examination study (for details of the methods, see http://www.terveys2000.fi/indexe.html). Using these data, the metabolic syndrome was assessed with the criteria of European Group for the Study of Insulin Resistance (EGIR) modification of World Health Organization (WHO) criteria: diabetics and highest quartile of non-diabetics for fasting glucose were excluded. To fulfill the EGIR criteria for the metabolic syndrome, two of the following needed to be present: Fasting glucose of ≥6.1 mmol/l, elevated blood pressure (mean of systolic blood pressure measurements of ≥140 mmHg, or mean of diastolic blood pressure measurements of ≥90 mmHG, or medication for hypertension), triglycerides of ≥2.0 mmol/l or HDL of ≤1.0 mmol/l or lipid-lowering medication, waist circumference of ≥94 cm for men and that of ≥80 cm for women.

### Gene and SNP selection

*SIRT1* SNP selection was based on HapMap phase 3 data (http://www.hapmap.org/) and tagging was done using the Tagger program in the Haploview 4.2 software [19]. The linkage disequilibrium (LD) within the gene and 10 kb of their 5' and 3' flanking regions, i.e. 54 kb (chr10:69,304–69,358 kb, NCBI36/hg18 assembly), was used to select tag SNPs capturing most of the genetic variation. The aim was to capture all the SNPs having a minor allele frequency (MAF) of >5% in the European population (CEU and TSI) in the HapMap database. The pair-wise r^2^ was set to ≥0.9 in order to select a tag SNP among the SNPs that were in LD. Four out of 19 *SIRT1* SNPs fulfilled the criterion, and three SNPs (rs3758391, rs2273773, rs17454621) were successfully included in the genotyping multiplex.

### Genotyping

Genomic DNA was isolated from whole blood according to standard procedures. The SNPs were genotyped at the Institute for Molecular Medicine Finland, Technology Centre, University of Helsinki using the MassARRAY iPLEX method (Sequenom, San Diego, CA, USA) [20], with excellent success (>95%) and accuracy (100%) rates [21]. For quality control purposes, positive (CEPH) and negative water controls were included in each 384-plate. Genotyping was performed blind to phenotypic information.

440 of 5910 individuals were removed due to a high missing genotype rate (i.e. >0.1). The total genotyping rate in the remaining individuals was 0.996. Finally, there were 5470 individuals and three *SIRT1* SNPs for the statistical analyses.

### Statistical analyses

Statistical analyses were performed using logistic or linear regression and additive genetic model. Unadjusted, age and sex adjusted, and age, sex and BMI adjusted models were calculated using PLINK software v1.07 [22]. The values presented in the text are from the age and sex adjusted models. Haplotype blocks were defined using Haploview software [19] and the confidence interval algorithm. For the continuous phenotypes (GSS, BMI, waist circumference, diastolic and systolic blood pressure, blood glucose, insulin resistance index, beta-cell index, LDL, total cholesterol, HDL, insulin, triglycerides) 10,000 permutations were used to produce empirical p-values in order to relax the assumption of normality. The p-values were corrected for multiple testing with the Bonferroni method by taking into account the number of SNPs and independent phenotypes. After the Bonferroni correction, p-values of <0.0056 are significant for seasonality, p<0.0071 for metabolic syndrome and p<0.0029 for health examination measurements, HOMAs, and diagnoses of type 2 and type 1 diabetes. For replication of the previous findings reported in the literature, i.e. depressive and anxiety disorders, p-values of <0.05 were considered significant. Population stratification was not addressed.

## Results

The participants’ general characteristics are reported in Table 1. The study population of 5910 subjects was 55.4% women and had a mean age of 53.1 years (SD = 15.0), BMI of 27.0 (SD = 4.7), GSS of 5.0 (SD = 3.0), blood pressure of 81.7/134.9 (SD = 11.3/21.3). 8.2% had depressive disorder, 5.3% had anxiety disorder, 23.2% had metabolic syndrome (EGIR). Most participants presented seasonal variations in sleep length, social activity, mood and energy level.

**Table 1:** General characteristics of the participants. MDD; major depressive disorder. HDL; High-density lipoprotein cholesterol. BMI; body mass index. LDL; Low-density lipoprotein cholesterol. GSS; global seasonality score.

			RS3758391						RS2273773						RS17454621															
	All		*CC*		*CT*		*TT*		*CC*		*CT*		*TT*		*C C*		*T C*		*T T*											
	n	%	N	%	n	%	n	%	n	%	n	%	n	%	n	%	n	%	n	%										
Gender																														
Female	3033	55.4	1173	38.8	1405	46.5	446	14.7	52	1.7	648	21.4	2324	76.9	29	1.0	511	16.9	2491	82.2										
Male	2437	44.6	872	35.8	1176	48.3	386	15.9	43	1.8	532	21.9	1858	76.4	11	0.5	398	16.4	2022	83.2										
MDD																														
Cases	249	6.5	84	33.9	120	48.4	44	17.7	1	0.4	51	20.5	197	79.1	2	0.8	43	17.3	204	81.9										
Controls	3597	93.5	1360	37.9	1691	47.1	538	15.0	66	1.8	776	21.6	2744	76.5	23	0.6	596	16.6	2970	82.8										
Dysthymia																														
Cases	117	3.2	38	32.5	55	47.0	24	20.5	0	0.0	28	24.1	88	75.9	1	0.9	18	15.4	98	83.8										
Controls	3597	96.8	1360	37.9	1691	47.1	538	15.0	66	1.8	776	21.6	2744	76.5	23	0.6	596	16.6	2970	82.8										
Depressive disorder (MDD and/or dysthymia)																														
Cases	323	8.2	108	33.5	154	47.8	60	18.6	1	0.3	71	22.0	250	77.6	3	0.9	51	15.8	269	83.3										
Controls	3597	91.8	1360	37.9	1691	47.1	538	15.0	66	1.8	776	21.6	2744	76.5	23	0.6	596	16.6	2970	82.8										
Panic disorder																														
Cases	97	2.6	36	37.1	45	46.4	16	16.5	0	0.0	22	22.7	75	77.3	1	1.0	12	12.4	84	86.6										
Controls	3597	97.4	1360	37.9	1691	47.1	538	15.0	66	1.8	776	21.6	2744	76.5	23	0.6	596	16.6	2970	82.8										
Social fobia																														
Cases	49	1.3	19	38.8	26	53.1	4	8.2	0	0.0	10	20.4	39	79.6	0	0.0	10	20.4	39	79.6										
Controls	3597	98.7	1360	37.9	1691	47.1	538	15.0	66	1.8	776	21.6	2744	76.5	23	0.6	596	16.6	2970	82.8										
Agoraphobia																														
Cases	26	0.7	4	15.4	16	61.5	6	23.1	0	0.0	8	30.8	18	69.2	0	0.0	7	26.9	19	73.1										
Controls	3597	99.3	1360	37.9	1691	47.1	538	15.0	66	1.8	776	21.6	2744	76.5	23	0.6	596	16.6	2970	82.8										
Generalized anxiety disorder																														
Cases	64	1.7	20	31.3	36	56.3	8	12.5	2	3.1	13	20.3	49	76.6	0	0.0	12	18.8	52	81.3										
Controls	3597	98.3	1360	37.9	1691	47.1	538	15.0	66	1.8	776	21.6	2744	76.5	23	0.6	596	16.6	2970	82.8										
Anxiety disorder not otherwise specified																														
Cases	202	5.3	68	33.7	104	51.5	30	14.9	2	1.0	50	24.8	150	74.3	1	0.5	35	17.3	166	82.2										
Controls	3597	94.7	1360	37.9	1691	47.1	538	15.0	66	1.8	776	21.6	2744	76.5	23	0.6	596	16.6	2970	82.8										
Type 1 diabetes																														
Cases	31	0.6	15	48.4	15	48.4	1	3.2	0	0.0	10	32.3	21	67.7	0	0.0	6	19.4	25	80.6										
Controls	5133	99.4	1915	37.4	2422	47.3	785	15.3	87	1.7	1106	21.6	3927	76.7	39	0.8	853	16.6	4233	82.6										
Type 2 diabetes																														
Cases	300	5.5	111	37.1	143	47.8	45	15.1	7	2.3	64	21.3	229	76.3	1	0.3	49	16.3	250	83.3										
Controls	5133	94.5	1915	37.4	2422	47.3	785	15.3	87	1.7	1106	21.6	3927	76.7	39	0.8	853	16.6	4233	82.6										
Metabolic syndrome (EGIR criteria)																														
Cases	1172	23.2	468	40.0	536	45.8	166	14.2	16	1.4	253	21.6	901	77.0	7	0.6	199	17.0	964	82.4										
Controls	3885	76.8	1416	36.5	1849	47.7	611	15.8	69	1.8	835	21.6	2970	76.7	30	0.8	643	16.6	3206	82.7										
Seasonal variation in sleep lenght																														
Cases	3978	73.8	1485	37.4	1873	47.2	613	15.4	66	1.7	853	21.5	3049	76.8	35	0.9	661	16.6	3276	82.5										
Controls	1409	26.2	534	38.0	668	47.5	203	14.4	26	1.8	306	21.8	1074	76.4	5	0.4	237	16.8	1165	82.8										
Seasonal variation in social activity																														
Cases	3796	71.9	1415	37.3	1803	47.6	573	15.1	63	1.7	817	21.6	2907	76.8	27	0.7	642	16.9	3121	82.3										
Controls	1487	28.1	554	37.4	700	47.3	227	15.3	26	1.8	322	21.7	1135	76.5	12	0.8	234	15.8	1239	83.4										
Seasonal variation in mood																														
Cases	4059	76.0	1516	37.4	1928	47.6	610	15.0	65	1.6	875	21.6	3110	76.8	33	0.8	688	17.0	3332	82.2										
Controls	1280	24.0	483	37.9	595	46.7	196	15.4	28	2.2	273	21.4	975	76.4	7	0.5	202	15.8	1069	83.6										
Seasonal variation in weight																														
Cases	2637	49.4	981	37.3	1242	47.2	410	15.6	34	1.3	541	20.6	2052	78.1	20	0.8	435	16.5	2179	82.7										
Controls	2698	50.6	1022	38.0	1271	47.2	398	14.8	58	2.2	611	22.7	2026	75.2	19	0.7	450	16.7	2224	82.6										
Seasonal variation in appetite																														
Cases	2282	42.6	832	36.6	1081	47.5	363	15.9	35	1.5	482	21.2	1758	77.3	26	0.8	503	16.3	2548	82.8										
Controls	3081	57.4	1180	38.4	1447	47.0	449	14.6	57	1.9	676	22.0	2342	76.2	14	0.6	390	17.1	1874	82.3										
Seasonal variation in energy level																														
Cases	4035	75.4	1494	37.1	1924	47.8	611	15.2	68	1.7	867	21.5	3091	76.8	32	0.8	684	17.0	3313	82.2										
Controls	1318	24.6	511	38.9	604	46.0	198	15.1	23	1.8	289	22.0	1002	76.3	8	0.6	211	16.0	1097	83.4										
High fasting glucose																														
Cases	820	15.0	314	38.3	383	46.8	122	14.9	20	2.4	187	22.8	612	74.7	9	1.1	138	16.8	672	82.1										
Controls	4646	85.0	1727	37.3	2198	47.4	710	15.3	75	1.6	993	21.4	3566	77.0	31	0.7	771	16.6	3837	82.7										
Elevated blood pressure or medication for hypertension																														
Cases	2603	47.7	984	37.9	1223	47.1	390	15.0	45	1.7	590	22.7	1961	75.5	14	0.5	430	16.5	2155	82.9										
Controls	2854	52.3	1058	37.1	1349	47.4	441	15.5	50	1.8	583	20.5	2215	77.8	26	0.9	478	16.8	2346	82.3										
High triglycerides or low HDL or lipid-lowering medication																														
Cases	1905	34.9	701	36.9	900	47.3	301	15.8	31	1.6	418	22.0	1453	76.4	10	0.5	325	17.1	1563	82.3										
Controls	3561	65.1	1340	37.7	1681	47.3	531	14.9	64	1.8	762	21.5	2725	76.7	30	0.8	584	16.4	2946	82.8										
Long waist circumference																														
Cases	3702	68.5	1401	37.9	1730	46.8	563	15.2	59	1.6	803	21.8	2829	76.6	26	0.7	625	16.9	3045	82.4										
Controls	1706	31.5	620	36.4	821	48.2	261	15.3	34	2.0	365	21.4	1305	76.6	14	0.8	273	16.0	1418	83.2										
	All			RS3758391									RS2273773									RS17454621								
				*CC*			*CT*			*TT*			*CC*			*CT*			*TT*			*C C*			*T C*			*T T*		
	n	mean	SD	n	mean	SD	N	mean	SD	n	mean	SD	n	mean	SD	n	mean	SD	n	mean	SD	n	mean	SD	n	mean	SD	n	mean	SD
Age	5470	53.1	15.0	2045	52.8	15.0	2581	53.5	15.0	832	52.7	15.1	95	55.4	16.5	1180	53.6	14.9	4182	52.9	15.0	40	52.1	14.6	909	53.2	15.0	4513	53.1	15.1
BMI	5454	27.0	4.7	2039	27.0	4.6	2574	27.0	4.7	829	27.0	4.8	94	26.1	4.6	1175	27.0	4.7	4172	27.0	4.7	40	25.5	3.8	907	27.0	4.8	4499	27.0	4.7
Waist circumference	5409	92.9	13.3	2022	92.8	13.3	2551	92.9	13.3	824	93.0	13.4	93	91.0	12.5	1168	93.0	13.2	4135	92.9	13.4	40	87.5	11.4	898	92.9	13.4	4464	92.9	13.3
Systolic blood pressure	5453	134.9	21.3	2041	134.8	21.5	2570	135.1	21.2	830	134.5	20.9	95	136.2	23.7	1173	136.6	21.7	4172	134.4	21.1	40	127.2	17.5	907	135.1	21.5	4498	135.0	21.2
Diastolic blood pressure	5451	81.7	11.3	2041	81.7	10.9	2569	81.7	11.6	829	81.9	11.2	95	81.8	11.6	1173	82.9	11.4	4170	81.4	11.2	40	80.3	8.1	907	81.6	10.9	4496	81.7	11.4
GSS	5206	5.0	3.0	1945	5.0	3.0	2468	5.1	3.1	782	5.1	3.0	89	4.5	2.9	1119	5.0	3.1	3985	5.0	3.0	38	5.1	2.5	862	5.1	3.1	4298	5.0	3.0
Insulin resistance index	5343	2.5	5.7	1999	2.7	8.2	2520	2.5	3.6	812	2.2	2.5	89	2.3	3.9	1157	2.5	4.1	4084	2.6	6.1	37	2.6	4.6	890	2.8	10.2	4408	2.5	4.2
Beta-cell index	5335	94.7	138.8	1992	94.4	88.5	2519	93.8	141.8	812	98.0	212.3	89	77.3	60.0	1156	95.3	181.3	4077	94.9	125.8	37	78.4	62.5	887	92.7	101.8	4403	95.2	145.7
fS-Glucose, mmol/l	5466	5.6	1.2	2041	5.6	1.3	2581	5.6	1.2	832	5.5	0.9	95	5.8	1.5	1180	5.6	1.2	4178	5.5	1.2	40	5.7	0.9	909	5.6	1.1	4509	5.6	1.2
fS-Cholesterol, mmol/l	5466	5.9	1.1	2041	6.0	1.1	2581	5.9	1.1	832	5.9	1.1	95	6.0	1.0	1180	6.0	1.1	4178	5.9	1.1	40	5.8	0.9	909	5.9	1.1	4509	5.9	1.1
fS-HDL, mmol/l	5466	1.3	0.4	2041	1.3	0.4	2581	1.3	0.4	832	1.3	0.4	95	1.3	0.4	1180	1.3	0.4	4178	1.3	0.4	40	1.4	0.3	909	1.3	0.4	4509	1.3	0.4
fS-LDL, mmol/l	5440	3.7	1.1	2035	3.7	1.1	2566	3.7	1.0	827	3.7	1.1	95	3.7	1.0	1174	3.7	1.1	4159	3.7	1.0	40	3.7	0.9	905	3.7	1.0	4487	3.7	1.1
fS-Triglycerides, mmol/l	5466	1.6	1.0	2041	1.6	0.9	2581	1.6	1.1	832	1.6	1.0	95	1.6	0.8	1180	1.6	1.1	4178	1.6	1.0	40	1.4	0.6	909	1.6	1.0	4509	1.6	1.0
fS-Insulin mU/l	5347	9.8	32.7	2002	9.8	18.1	2521	10.1	44.5	812	8.8	8.1	89	8.4	9.6	1157	9.2	8.7	4088	10.0	37.0	37	9.2	13.1	890	10.0	24.4	4412	9.7	34.2

**Fig 1:**
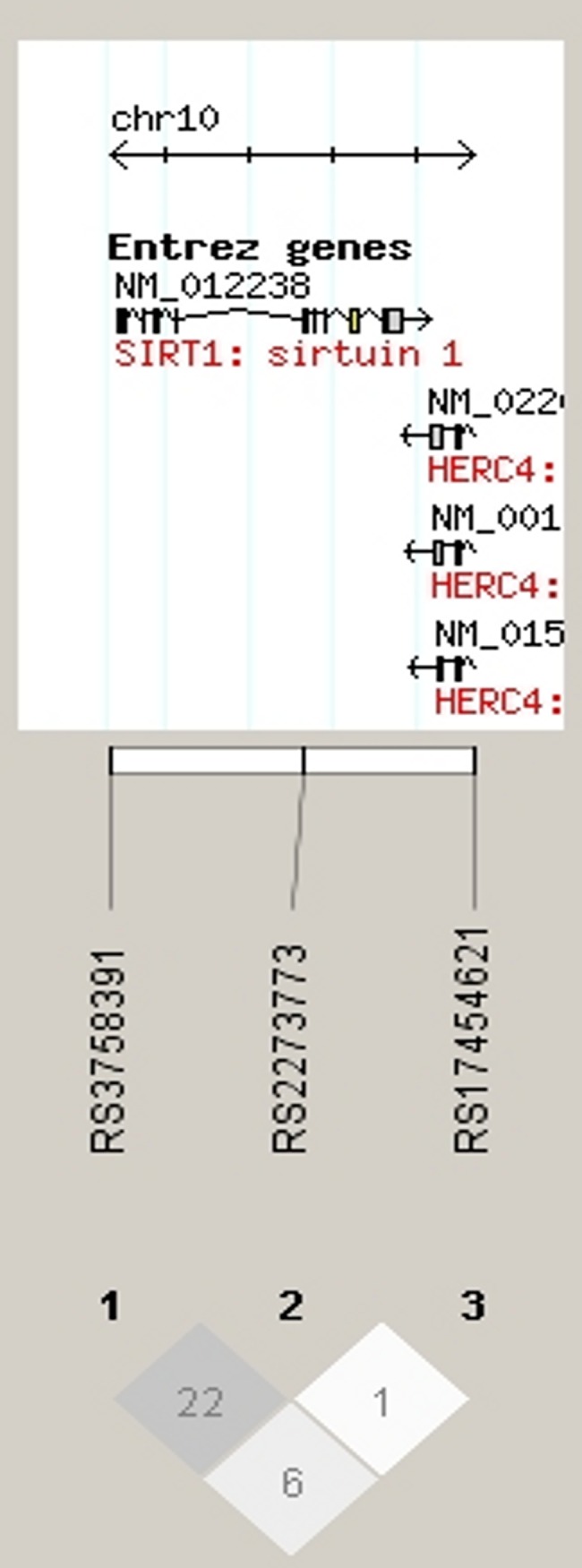
The *SIRT1* SNPs analyzed and their location showing r^2^ values. The confidence interval algorithm implemented in the Haploview program was used to construct the haplotype blocks.

Genotype and allele frequencies and the Hardy-Weinberg equilibrium estimates are shown in Table 2. No haplotype blocks were formed for *SIRT1* (Fig 1). All the SNP association results are shown in S1 Table. *SIRT1* rs3758391 *T* allele showed nominally significant associations with depressive disorders (OR = 1.19, 95% CI of 1.01 to 1.40, p = 0.040, see Table 3), metabolic syndrome (OR = 0.88, 95% CI of 0.80 to 0.97, p = 0.01, see Table 3), insulin resistance index (beta = -0.26, 95% CI of -0.48 to -0.04, p = 0.019, empirical p = 0.02, see Table 3) and blood glucose (beta = -0.05, CI of -0.09 to -0.002, p = 0.04, empirical p = 0.04, Table 3). The associations with metabolic syndrome, insulin resistance index and blood glucose did not remain significant after correcting for multiple testing.

**Table 2:** *SIRT1* genotype counts and frequencies and Hardy-Weinberg equilibrium p-values. BP; Base pair position. A1; Minor allele. A2; Major allele. MAF; Minor allele frequency. A1A1, A1A2, A2A2; Genotype counts and frequencies (%). P; Hardy-Weinberg p-value

SNP	BP (NCBI36/hg18)	A1	A2	MAF	A1A1 (%)	A1A2 (%)	A2A2 (%)	P
rs3758391	69313348	*T*	*C*	38.9	832 (15.2)	2581 (47.3)	2045 (37.5)	0.71
rs2273773	69336604	*C*	*T*	12.6	95 (1.7)	1180 (21.6)	4182 (76.6)	0.27
rs17454621	69356812	*C*	*T*	9.1	40 (0.7)	909 (16.6)	4513 (82.6)	0.51

**Table 3:** Results (P/EMP<0.05) of the *SIRT1* SNP associations (unadjusted on the first line / age and sex adjusted on the second line / age, sex and BMI adjusted on the third line). A1; Tested allele (minor allele). N; Number of genotypes for the phenotype. L95, U95; Lower and upper bounds of 95% confidence interval for odds ratio. P/EMP: p-value / empirical p-value

Phenotype	SNP	A1	N	OR/beta	L95	U95	P	EMP
Depressive and anxiety disorders								
Depressive disorders	RS3758391	*T*	3911	1.18	1.00	1.39	0.05	
				1.19	1.01	1.40	0.04	
				1.19	1.01	1.40	0.04	
Metabolic syndrome (EGIR) and its components								
Metabolic syndrome	RS3758391	*T*	5046	0.90	0.82	0.99	0.03	
				0.88	0.80	0.97	0.01	
				0.86	0.77	0.96	0.01	
Seasonal variations in mood and behavior								
Weight	RS2273773	*C*	5322	0.84	0.75	0.95	0.003	
				0.85	0.76	0.95	0.01	
				0.86	0.76	0.97	0.01	
Health examination measurements, HOMAs, and diagnoses of type 2 and type 1 diabetes.								
Diastolic blood pressure	RS2273773	*C*	5438	1.13	0.49	1.76	0.001	0.001
				1.06	0.43	1.68	0.001	0.001
				1.23	0.63	1.82	0.0001	0.0003
Systolic blood pressure	RS2273773	*C*	5440	1.88	0.68	3.08	0.002	0.003
				1.23	0.19	2.28	0.02	0.02
				1.46	0.45	2.48	0.005	0.004
Insulin resistance index	RS3758391	*T*	5331	-0.25	-0.47	-0.03	0.03	0.02
				-0.26	-0.48	-0.04	0.02	0.02
				-0.28	-0.49	-0.06	0.01	0.01
Blood glucose	RS3758391	*T*	5454	-0.04	-0.09	0.009	0.11	0.11
				-0.05	-0.09	-0.002	0.04	0.04
				-0.05	-0.09	-0.002	0.04	0.04

The association of *SIRT1* rs2273773 with the seasonal variation in weight (OR = 0.85, 95% CI of 0.76 to 0.95, p = 0.005) remained significant after the Bonferroni correction, the *C*-allele being associated with the decreased odds for the seasonal variation in weight (Table 3). *SIRT1* rs2273773 *C* allele associated with both high diastolic (beta = 1.06, 95% CI of 0.43 to 1.68, p = 0.001, empirical p-value = 0.001) and systolic blood pressure (beta = 1.23, 95% CI of 0.19 to 2.28, p = 0.02, empirical p-value = 0.02), of which the association with diastolic blood pressure remained significant after the Bonferroni correction, the *C*-allele having the odds for higher diastolic blood pressure.

## Discussion

Our current results from the population-based health examination study suggested the minor *C*-allele of synonymous (Leu→Leu) *SIRT1* rs2273773 polymorphism to contribute to higher diastolic blood pressure, and to protect from seasonal variation in body weight. However, the SNP showed no evidence of association with BMI or the metabolic syndrome or its components, as assessed with the EGIR modification of WHO criteria. In agreement, *CC* carriers have previously been reported to have high systolic and diastolic blood pressures in men [23], and no association with metabolic syndrome in morbidly obese subjects has been found [24]. However, the *C*-allele (or *CC* genotype or *C* carriers) has been reported to be protective against cardiovascular diseases [25] and contribute to higher energy expenditure [26], a lower BMI [27], and lower fasting glucose concentrations and body fat ratios in men [23]. Moreover, the T-allele of *SIRT1* rs2273773 was seen, as part of two haplotypes of *SIRT1*, to be associated with schizophrenia but not with bipolar disorder [10]. We were not able to test this association, since these disorders were not assessed with the method used for diagnostic interview in our study. In addition, our study provides further support of the association between *SIRT1* (rs3758391) and depressive disorders (major depressive disorder and dysthymia).

Our study does not come without limitations. The assessment of the seasonal variations in mood and behavior was based on the self-report only and only limited variables were controlled for in the statistical analysis. On the other hand, there are strengths in our study such as the number of participants enrolled from a nation-wide and representative sample of the adult general population aged 30 years and older, the health examination protocol for the assessment of the metabolic syndrome, the diagnostic interview for the assessment of depressive and anxiety disorders, and the coverage of *SIRT1* for the assessment of genetic association.

In conclusion, we found that *SIRT1* (rs2273773) accounts for the seasonal variation in body weight. In addition, our study gave further support for the role of *SIRT1* in depressive disorders (rs3758391) and diastolic blood pressure (rs2273773). Thus, *SIRT1* appears to contribute to seasonal, mood and cardiovascular physiology in humans.

## Supporting Information

S1 Table All results of the *SIRT1* SNP associations. (XLSX)
